# Synthesis of Gold Nanoparticles with Buffer-Dependent Variations of Size and Morphology in Biological Buffers

**DOI:** 10.1186/s11671-016-1290-3

**Published:** 2016-02-04

**Authors:** Syed Rahin Ahmed, Sangjin Oh, Rina Baba, Hongjian Zhou, Sungu Hwang, Jaebeom Lee, Enoch Y. Park

**Affiliations:** Research Institute of Green Science and Technology, Shizuoka University, 836 Ohya Suruga-ku, Shizuoka, 422-8529 Japan; Department of Cogno-Mechatronics Engineering, Pusan National University, Busan, 46279 South Korea; Department of Applied Biological Chemistry, Shizuoka University, 836 Ohya Suruga-ku, Shizuoka, 422-8529 Japan; Institute of Solid State Physics, Chinese Academy of Sciences, Hefei, 230031 P. R. China; Department of Nanomechatronics Engineering, Pusan National University, Miryang, 627-706 South Korea; Graduate School of Science and Technology, Shizuoka University, 836 Ohya Suruga-ku, Shizuoka, 422-8529 Japan

**Keywords:** Gold nanoparticles, Good’s buffer, Synthesis route, MD simulation, Cell viability

## Abstract

The demand for biologically compatible and stable noble metal nanoparticles (NPs) has increased in recent years due to their inert nature and unique optical properties. In this article, we present 11 different synthetic methods for obtaining gold nanoparticles (Au NPs) through the use of common biological buffers. The results demonstrate that the sizes, shapes, and monodispersity of the NPs could be varied depending on the type of buffer used, as these buffers acted as both a reducing agent and a stabilizer in each synthesis. Theoretical simulations and electrochemical experiments were performed to understand the buffer-dependent variations of size and morphology exhibited by these Au NPs, which revealed that surface interactions and the electrostatic energy on the (111) surface of Au were the determining factors. The long-term stability of the synthesized NPs in buffer solution was also investigated. Most NPs synthesized using buffers showed a uniquely wide range of pH stability and excellent cell viability without the need for further modifications.

**Electronic supplementary material:**

The online version of this article (doi:10.1186/s11671-016-1290-3) contains supplementary material, which is available to authorized users.

## Background

Gold nanoparticles (Au NPs) are one of the most intensely studied materials in the field of nanotechnology. Recent research interest in Au NPs has stemmed from their peculiar localized surface plasmon resonance (SPR) behavior as well as their size- and shape-dependent physicochemical characteristics, properties that are not observed in bulk Au [1–3]. The surface chemistry of Au NPs permits binding with thiols and amines [4, 5], allowing for easy tagging of the NPs with various proteins and biomolecules. These properties have led to important biomedical applications including selective targeting [6–8], cellular imaging [9, 10], and biosensing [11–14]. Although Au NPs are commonly believed to be chemically inert, recent evidence reveals their high catalytic activity that emerges at significantly reduced particle sizes [15–17], and more importantly, with deliberately tailored shapes introducing high-index facets on the NP surfaces. Au NPs are required in many commercial and industrial applications, with novel emerging applications dramatically increasing global demand for Au NPs. These uses include biomolecule- and/or biopolymer-conjugated Au NPs acting as bio-markers and bio-delivery vehicles in medicine and pharmaceuticals, as anti-aging components in cosmetic products [18–20], for permanent coloration of valuable wool or cotton textiles [21], as novel coatings and paints created from polymer/gold nanocomposites [22], as non-volatile memory devices and metal printing inks in electronics [23, 24], and as substrates in surface-enhanced Raman scattering studies [25, 26]. Au NPs do not affect the functional activity of biomolecules after binding, which in turn can be used for the detection of specific target analytes [27]. Because of all the above listed advantages, Au NPs are used in the development of lateral flow assays, a one-step in situ screening test for the analyte.

In particular, more attention must be given to completely biocompatible and agglomeration-free syntheses of Au NPs with diverse biomedical applications in order to meet the increasing demand. Current Au NP preparations are limited by phase transfer, as surface modification causes difficulty in transferring Au NPs to a designated solvent. Many applications of Au NPs require the particles’ introduction to biological buffer systems, and researchers have often failed to transfer Au NPs from their aqueous system to physiological media due to the NPs’ instability, seriously limiting their therapeutic and diagnostic applications [28, 29]. In this process, Au NPs typically lose their original plasmonic band, becoming dark brown precipitates. The ability to develop simple, reliable, and accessible methods to efficiently control and manipulate Au NP size and morphology in physiological media remains one of the most important endeavors in this field of research.

Organic biological buffers can advantageously replace mineral buffers in many biological research and analysis applications. Several biological buffers such as PIPES, MES, and TAPSO are useful for in vitro cell culturing, enzyme assays, protein crystallization (bicine), medicine (triethanolamine, TEA), and some electrophoretic applications (e.g., bis-tris propane, TAPS) (see the “Methods” section to find full names of buffers). Universally applicable buffers for biochemistry must be water-soluble and should not produce chelates or possess complex-forming tendencies with metal ions. However, no buffer is truly and completely inert in biological systems. For example, the Good’s buffers that contain piperazine rings (such as PIPES) can generate nitrogen-centered free radicals. Some buffers have also shown significant affinities with metal ions [30], resulting in the formation of metal complexes with appreciable association constants. While Habib et al. [31] reported the successful synthesis of Au NPs in MES and HEPES buffers, fine-tuning of the structures and sizes of the NPs remains an ongoing challenge, and the study of Au NP formation with different buffers is also necessary.

In this study, we have developed 11 synthetic methods to obtain Au NPs, 10 of which were developed using biological buffers. Here, all reagents used in these syntheses also acted as reducing and particle-stabilizing agents during synthesis. It is interesting to note that no additional chemicals were required for further stabilization of these NPs. Molecular dynamics (MD) simulations and electrochemical analysis were performed in order to better understand the surface stability of Au NPs co-functionalized with different reagents. We also characterized the resulting Au NPs and discussed their properties.

## Methods

### Materials

**Fig. 1:**
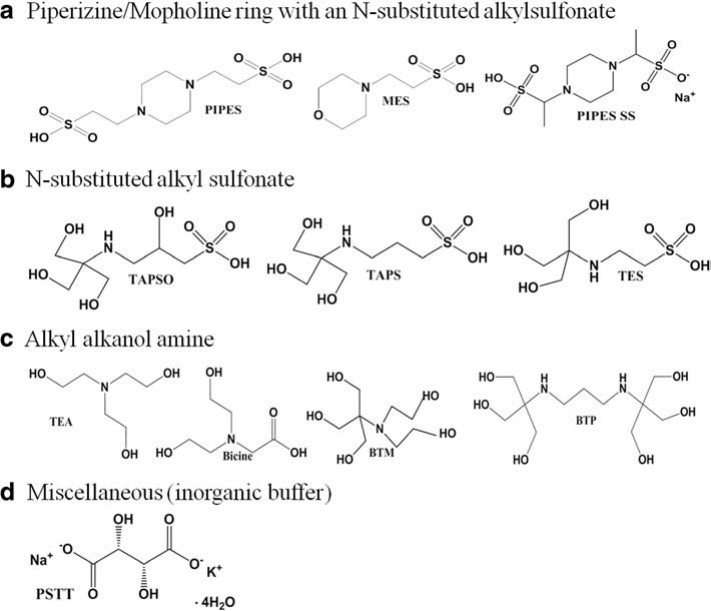
Molecular structures of buffers used. It is divided into four groups: **a**. piperizine/mopholine ring with an N-substituted alkyl sulfonate, **b** N-substituted alkyl sulfonate, **c** alkyl alkanol amine, and **d** miscellaneous (inorganic buffer)

Abbreviated and full names of each buffer are as follows : 1,4-piperazinediethanesulfonic acid (PIPES), 2-(*N*-morpholino) ethanesulfonic acid (MES), 3-{[1,3-dihydroxy-2-(hydroxymethyl)propan-2-yl]amino}-2-hydroxypropane-1-sulfonic acid (TAPSO), 2-{[1,3-dihydroxy-2-(hydroxymethyl)propan-2-yl]amino}ethanesulfonic acid (TES), 3-{[1,3-dihydroxy-2-(hydroxymethyl)propan-2-yl]amino}propane-1-sulfonic acid (TAPS), 2-[bis(2-hydroxyethyl)amino]-2-(hydroxymethyl)propane-1,3-diol (bis-tris methane or BTM), tris(2-hydroxyethyl)amine (TEA), 2-[bis(2-hydroxy)ethylamino]acetic acid (bicine), 2,2'-(1,3-Propanediyldiimino)bis[2-(hydroxymethyl)-1,3-propanediol] (bis-tris propane or BTP) buffers (Fig. 1) were purchased from TCI Tokyo Chem. Co. (Tokyo, Japan); PIPES sesquisodium salt (PIPES-SS) and potassium sodium tartrate tetrahydrate (PSTT) were purchased from Wako Pure Chem. Inc. (Osaka, Japan). The buffers were classified according to their primary effective functional groups: A. piperizine/mopholine ring with an N-substituted alkyl sulfonate, B. N-substituted alkyl sulfonate, C. alkyl alkanol amine, and D. miscellaneous (inorganic buffer). All experiments were performed using high-purity deionized (DI) water with an electrical conductivity >18 MΩ.

### Synthesis of Au NPs Using Different Buffers

Each synthesis was performed in a 50-mL conical flask under ambient conditions. The flask was washed using King’s solution and a large volume of DI water prior to each synthesis. The concentration of Au, reaction temperature, and reaction time were varied depending on the reducing ability of the buffer used in each process. In these experiments, NP syntheses using mixtures of two or three different buffers were not considered, as these may give more complicated results that could not be explained using current theoretical understanding and simulations, owing to the many electrolytes present in a solution of even a single buffer. In addition, there is a lack of biological or chemical research employing combined buffer solutions for practical reasons. The detail of the synthesis using different buffers is as follows.

### PIPES Buffer

Four milliliters of 100 mM aqueous PIPES buffer and 1 mL of 20 mM aqueous HAuCl_4_ were placed in 36 mL DI water and vigorously stirred at 25 °C. The solution turned deep pink within 1 min, indicating particle formation, where the buffer serves as both reducing and capping agent without heating or cooling.

### MES Buffer

Four milliliters of 100 mM aqueous MES buffer and 1 mL of 20 mM aqueous HAuCl_4_ were placed in 36 mL DI water under vigorous stirring at 100 °C for 10 min. The deep pink solution was then cooled while stirring to obtain Au NPs.

### PIPES-SS Buffer

One milliliter of 20 mM aqueous HAuCl_4_ and 4 mL of 100 mM aqueous PIPES-SS buffer were placed in 36 mL DI water under vigorous stirring at 150 °C for 10 min. The solution turned light orange within 5 min, indicating particle formation.

### TAPSO Buffer

One milliliter of 20 mM aqueous HAuCl_4_ was mixed with 36 mL DI water and boiled at 100 °C for 5 min. Then, 4 mL of 0.1 M aqueous TAPSO buffer was added, and the particles formed within 30 s.

### TAPS Buffer

One milliliter of 20 mM aqueous HAuCl_4_ was mixed with 36 mL DI water and boiled at 100 °C for 5 min. Then, 4 mL of 0.5 M aqueous TAPS buffer was added; the reaction was allowed to continue for 1 h.

### TES Buffer

One milliliter of 20 mM aqueous HAuCl_4_ was mixed with 36 mL DI water and boiled at 100 °C for 5 min. Then, 4 mL of 0.05 M aqueous TES buffer was added, and the reaction continued for 10 min.

### TEA Buffer

One milliliter of 20 mM aqueous HAuCl_4_ and 4 mL of 100 mM aqueous TEA buffer were placed in 36 mL DI water under vigorous stirring at 25 °C. The solution turned deep blue within 30 s, indicating particle formation.

### Bicine Buffer

One milliliter of 20 mM aqueous HAuCl_4_ was mixed with 36 mL DI water and boiled at 100 °C for 5 min. Then, 4 mL of 0.05 M aqueous bicine buffer was added, and the reaction was allowed to continue for 1 min.

### BTM Buffer

One milliliter of 20 mM aqueous HAuCl_4_ and 4 mL of 100 mM aqueous bis-tris methane buffer were placed in 36 mL DI water under vigorous stirring at 25 °C. The solution turned a deep pink color within 30 s, indicating particle formation.

### BTP Buffer

One milliliter of 20 mM aqueous HAuCl_4_ and 4 mL of 100 mM and aqueous bis-tris propane buffer were placed in 36 mL DI water under vigorous stirring at 100 °C for 5 min. The solution turned light pink within 5 min, indicating particle formation.

### PSTT

One milliliter of 20 mM aqueous HAuCl_4_ was placed in 36 mL DI water under vigorous stirring at 150 °C for 20 min. Then, 4 mL of 100 mM aqueous PSTT was added with continued stirring. The solution turned light pink within 5 min, indicating particle formation.

### pH Stability, Spectroscopic, and Microscopic Analysis of Synthesized NPs

After synthesizing NPs using the above methods, aggregation tests were performed across a wide range of pH values. Aqueous Au NP solutions at different pH values were prepared by mixing adequate amounts of NH_4_OH in demineralized distilled water to form basic solutions (pH 8–9), whereas acidic media (pH 2–6) was prepared with HCl in water. The pH stability for each solution was monitored using a digital pH meter (Model HM-25R, DKK-TOA, Tokyo, Japan). Ultraviolet-visible absorption spectra were also collected (Infinite M 200, Tecan, Männedorf, Switzerland). Transmission electron microscopy (TEM) images were obtained at an acceleration voltage of 120 kV (TEM-1400, JEOL, Tokyo, Japan). Chemical reactions and surface functional groups were monitored using FT-IR spectroscopy (FT-IR 6300, JASCO Corp., Tokyo, Japan).

### Electrochemical Analysis of NP Synthesis

Electrochemical analyses were performed using cyclic voltammetry (IVIUM soft, Ivium Technology, Eindhoven, Netherlands) to monitor the reduction ability of the buffers. An aqueous solution of 0.1 M buffer was used for each measurement with 0.2 M sodium sulfate as a supporting electrolyte. The working electrode was a glassy carbon (GC) electrode, which was polished with 0.3 μm alumina powder on soft lapping pads and deionized water to achieve a mirror-like surface. An Ag/AgCl electrode (3.0 M NaCl) was used as the reference electrode, and a platinum wire was used as the counter electrode. All the electrodes were purchased from ALS Co., Ltd., Tokyo, Japan. The cell volume was 20 mL, and the scan rate was 0.1 V · s^−1^. Cyclic voltammograms were recorded in the potential range from −0.1 to 1.5 V. All measurements were performed at room temperature (the range of 20 to 25 °C) and ambient pressure.

### Computational Simulation of Buffer-Induced Synthesis of Au NPs

The interactions of buffers and their mixtures with the Au NPs were determined using computational simulations. The dimensions of the Au surface used for Au/buffer molecule interface simulations were 8.65 × 8.65 × 9.42 Å for Au (111). First, buffer models were built with the amorphous cell module in the material studio. A packing model featuring a density of 1.0 g cm^−3^ and containing one buffer molecule was constructed by the amorphous cell module. A 5000-step energy minimization was performed at the initial stage to eliminate undesirable contacts (e.g., overlapping or close contact). Molecular dynamic calculations were performed using three models in order to obtain a stable configuration, and the calculations were conducted for 100 ps at 298 K under the canonical ensemble (NVT) where the amount of substance is defined as *N*, volume as *V*, and temperature as *T*. These models were based on the experimental conditions for the reduction of Au NPs by the different buffer solutions.

### Biocompatibility Tests

Cytotoxicity evaluation of the Au NPs was performed using 3-(4,5-dimethylthiozol-2-yl)-2,5-diphenyl tetrazolium bromide (MTT) assay, as described by Mossman [32]. Approximately 2 × 10^3^ HEK 294T cells in their exponential growth phase were seeded in a flat-bottomed 96-well polystyrene-coated plate and incubated for 48 h at 37 °C in a 5 % CO_2_ incubator. After incubation, the medium was discarded and 100 μL fresh medium was added per well to the cells, after thorough washing with sterile phosphate-buffered saline (PBS). Next, 10 μL of Au NPs from each sample was added to the plate, in which the NP amount was selected from the previous optimization experiments for biocompatibility [33]. After 24 h of further incubation, 10 μL of MTT reagent was added to each well, and a final incubation period of 4 h progressed. The media were then discarded from the wells, and 200 μL dimethyl sulfoxide was added to solubilize the formazan crystals that had formed in the interim. Readings were recorded using a Bio-Rad enzyme-linked immunosorbent assay reader at 490 nm, with subtraction for plate absorbance at 530 nm.

## Results and Discussion

### Electrochemical Behavior

**Fig. 2:**
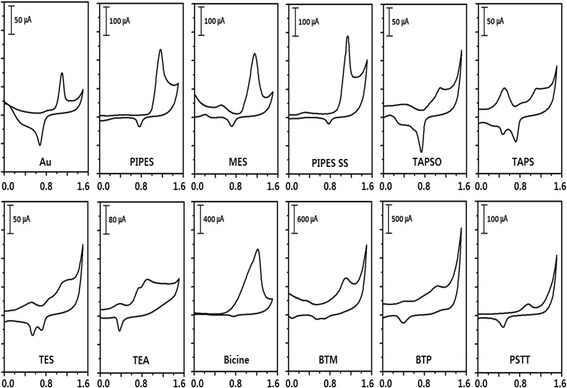
Cyclic voltammograms of gold and buffers in aqueous 0.2 M sodium sulfate electrolyte; scan rate was 0.1 V/s

The oxidation and reduction potentials of HAuCl_4_ and buffers were evaluated in 0.2 M sodium sulfate electrolyte (Fig. 2). In aqueous conditions, the reduction potential of HAuCl_4_ to Au^0^ was 0.60 V (vs. Ag/AgCl) while the oxidation potential of Au^0^ was 1.22 V (vs. Ag/AgCl). These values were compared to the reduction potentials of the various biological buffers used in this work. The buffers under study are divided into four groups based on the functional groups, as described previously: A; piperizine ring or morpholine ring with an N-substituted alkyl sulfonate, B; alkyl sulfonate, C; alkyl alkanolamine and D; inorganic buffer, i.e., PSTT. The peak oxidation potentials of the buffers are gathered in Table 1. Group A has one anodic peak at approximately 1.1 V, and a small cathodic peak could also be observed near +0.7 V vs. Ag/AgCl. In group B, TAPSO and TES have anodic peaks at 1.08 and 1.14 V, respectively. On the other hand, TAPS has two anodic peaks at 0.5 and 1.1 V. In group C, TEA has two anodic peaks at 0.73 and 0.89 V and one cathodic peak at 0.36 V. Bicine has one anodic peak at 1.2 V and no obvious cathodic peak. Bis-tris methane (BTM) and bis-tris propane (BTP) have one anodic peak at ~1.0 V. However, group D has one positive anodic peak at 0.95 and one cathodic peak at 0.48. When the oxidation peak falls within the range of potentials between the reduction and oxidation potentials of the gold system, HAuCl_4_ should be reduced thermodynamically. Oxidation potentials of all buffers were located between 0.6 and 1.22 V, which is a range that includes the redox potential of gold. Experimentally, this means that those buffers are predicted by electrochemistry to allow for Au NP formation. In addition, the two oxidation potential peaks of TAPS and TEA indicate that there are several oxidation steps in those redox systems, which could affect the formation of Au NPs in a different manner than the other buffers.

**Table 1:** Redox potentials of buffers in a 0.2 M sodium sulfate electrolyte solution

Group	Buffer	Reduction peak (V)	Oxidation peak (V)
A	PIPES	0.76	1.16
	MES	0.72	1.15
	PIPES-SS	0.76	1.13
B	TAPSO	0.73	1.08
	TAPS	0.47/0.72	0.50/1.11
	TES	0.54/0.72	0.55/1.14
C	TEA	0.36	0.73/0.89
	Bicine	–	1.2
	BTM	0.51/0.68	1.09
	BTP	0.39	1.04
D	PSTT	0.48	0.95

### UV-Visible Absorption Spectra of the Synthesized Au NPs

**Fig. 3:**
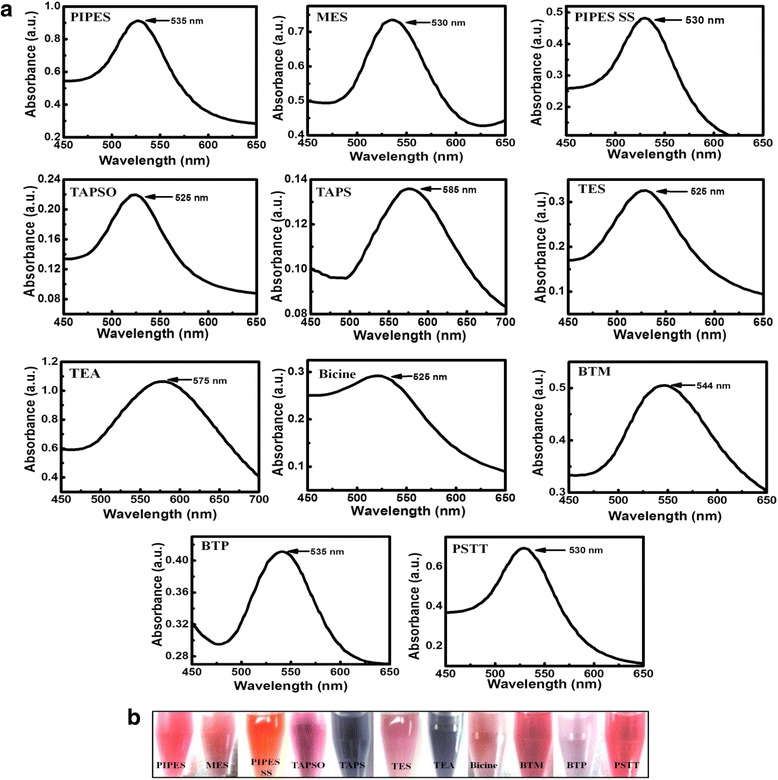
**a** UV-visible spectra and **b** solution photo images of Au NPs synthesized using different buffer solutions

Figure 3a shows the UV-visible spectra of Au NPs formed from the different solutions. Most spectra show a single absorption peak (*λ*_max_) in the visible range between 530 and 550 nm, indicating that the size and shape of the NPs are monodisperse. Since the reducing abilities of respective buffer molecules are different, additional adjustments in precursor concentration, reaction temperature, and duration were sometimes necessary. However, the Au NPs formed from TAPS present a strong band centered near 585 nm, and TEA presents an irregularly broad band, resulting from the composition of anisotropic-shaped nanomaterials with spherical NPs. Spherical Au NPs are known to show a strong absorption band in the visible region between 520 and 550 nm. This absorption, called the plasmon resonance absorption, originates from the collective oscillation of free electrons (in the case of Au, the 6s electrons of the conduction band) from the metallic atoms. The plasmon absorption band depends on the size, shape, and aggregation behavior of the NPs. For non-spherical Au NPs, however, plasmon resonance splitting can produce much broader absorption bands than the standard plasmon resonance peak (ca. 525 nm). A broader absorption band for the Au NPs was observed from the TAPS and TEA buffers, while MES and BTP presented sharper bands. In our experimental conditions, the band broadness can be determined primarily by the chemical reducibility and reaction temperature. Figure 3b presents images of Au NP solutions synthesized using different buffer solutions. Since the experiment was focused on a one-pot synthesis of wholly biocompatible NPs, the parameters with the greatest effect on shaping the NPs during nucleation and growth in each synthesis were not closely monitored and will be studied in future. However, it is expected that stronger reducibility and higher reaction temperatures induce the sharpers band and the smaller sizes of NPs produced under those conditions.

### Electron Microscopy Analysis of NP Morphology

**Fig. 4:**
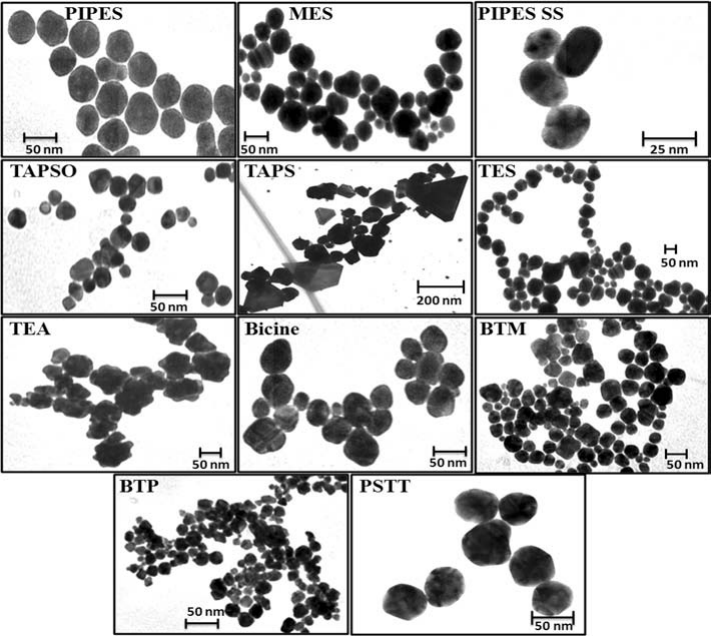
TEM images of Au NPs formed using different buffer solutions (images are not on the same size scale)

Figure 4 provides typical TEM images of the Au NPs formed in this study. Most NPs show roughly spherical structures, except those formed using TAPS and TEA buffers. The NPs formed by the reaction with TAPS buffer show a mixture of randomly dispersed shapes, including many different shapes that are not controlled, while the NPs obtained from the reaction with TEA show spiky structures. In terms of optical spectroscopy, NPs with these non-spherical geometric shapes are generally responsible for additional plasmon resonance absorption bands, as depicted by the broadened spectrum of the NPs formed in TEA. Plasmonic NPs containing spiky structures have also seen attention in the development of optical sensors [34]. Large scale TEM images of synthesized Au NPs (using MES, BTM and TEA) are available in Additional file 1: Figure S1–S3. The size and shape of NPs synthesized in this manner were monodisperse for the most part, which is one typical advantage of buffer-based NP synthesis. Since the buffers acted as both reducing and stabilizing agents during the synthesis, the high abundance of the buffer chemicals in the reaction helped improve the homogeneity of the reaction conditions in the reactor to keep a steady chemical state until reaction of the Au precursors went to completion.

In our buffer-based synthesis systems, these buffers displayed significant affinity to a number of metal ions, resulting in the formation of metal complexes. However, these buffer complexes are not stable and are readily oxidized by Au(III). This oxidation is accompanied by the simultaneous reduction of Au(III) to Au(II)/Au(I), and finally to Au(0), which results in the formation of Au NPs. NP formation can be completed within a few seconds to a few hours, depending on the buffer chemical, and the formed particles are stable for periods ranging from a few days to several months. BTM, containing five hydroxyl groups, was found to induce the spontaneous, instant formation of Au NPs at room temperature. The second fastest Au NP formations occurred when using the TEA and PIPES buffers. The three hydroxyl groups in TEA and the –SO_3_H groups and piperazine rings in PIPES and PIPES-SS were able to reduce Au(III) to Au(0) within a few minutes at room temperature. Note that this short reaction time may induce less size and shape homogeneity in the formed NPs, as it provides insufficient time to allow for Ostwald ripening. The other buffers contain either electron-donating groups or fewer electron-withdrawing groups, which result in slower oxidation reactions; these require heating at 100 °C to accelerate the formation of Au NPs. Unreacted buffer salts may introduce additional confusion to the research in this field. Additional file 1: Figure S4a depicts unreacted buffer salts with cubic structures. After washing and re-dispersing in water, these salts form urchin-like structures (Additional file 1: Figure S4b), which ultimately break into many small parts (Additional file 1: Figure S4e).

### Zeta Potential and pH Stability of Au NPs

**Fig. 5:**
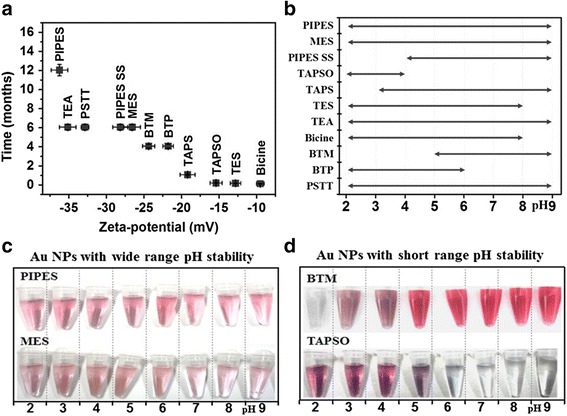
Physical stability of Au NPs: **a** zeta potential (*X*-axis) vs. stability at 4 °C (*Y*-axis); **b** pH stability of NPs; visualizations of Au NPs with wide-range (**c**) and-short range (**d**) pH stability when stored at 4 °C (10~20 mL)

The zeta potential of a NP solution is predictive of its long-term stability and resistance to aggregation at different pH values. NPs with a low zeta potential value will eventually aggregate due to Van der Waals forces. The correlation between the zeta potential and the stability (at 4 °C) of the synthesized Au NPs is shown in Fig. 5a. Of the Au NPs synthesized in this paper, NPs synthesized using the PIPES buffer have the highest zeta potential (−36.1 mV) and show the greatest stability due to the strong surface adsorption of the PIPES buffer onto the Au surface. However, the NPs synthesized using bicine, TES, and TAPSO all precipitated after only a short time period. The stability of the NPs was also examined across a wide range of pH values, as shown in Fig. 5b. The NPs showed clear single plasmonic peaks within this stable pH range, but outside it, the NPs exhibited considerably decreased absorbances or peak blue-shifting, most likely caused by the formation of agglomerates due to changes in surface charge. For example, Fig. 5c, d displays optical images of some Au NPs at different pH ranges divided into two groups: wide-range pH stability (5C) and short-range pH stability (5D). For wide-range pH stability Au NPs, the color did not change with the pH value (PIPES, MES as examples) whereas for short range pH stability Au NPs, a bluish color developed once the pH was adjusted outside of that stable range (BTM, TAPSO as examples).

### Binding Interaction of Reducing Agents and Au Surface by MD Simulation

**Fig. 6:**
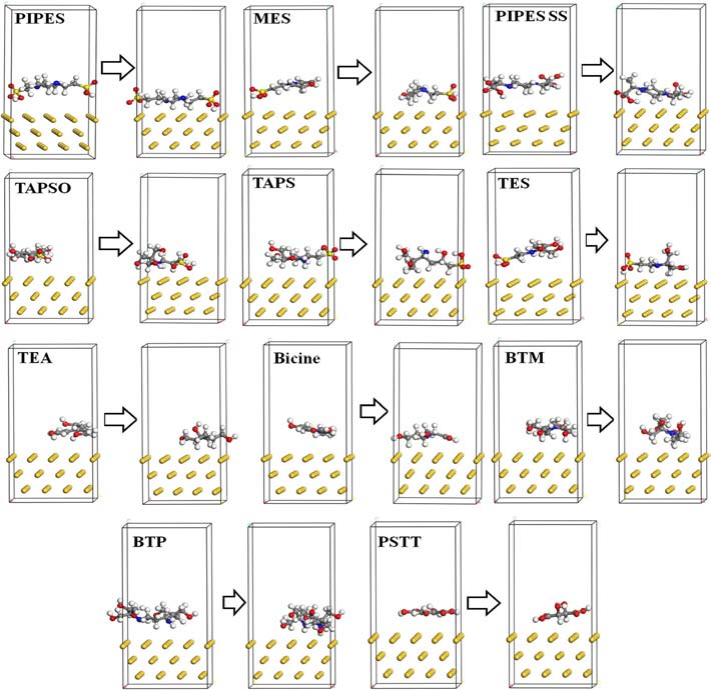
Molecular dynamic simulation: snapshots of reagent-Au models before (*left*) and after (*right*) MD simulation

The interaction energies between each reagent and the Au surface were studied using molecular dynamic (MD) simulations. Two layers of the slab structure with periodic boundary conditions were used to build the buffer-Au models, as shown in Fig. 6. The application of periodic boundary conditions made it possible to investigate the adsorption behavior of a reagent at a desired coverage on the Au surface. Such adsorption behavior can give information on the reagent’s stability; for example, higher adsorption of reagents on the Au surface implies that a strong interaction energy exists between the reagent and the Au surface, imparting the system with long-term stability. Here, a vacuum space of 10 Å was applied to separate the central slab and its periodic images, and a Au crystal was placed on one side of the slab. A 5000-step energy minimization was performed on the layer model at the initial stage. Molecular dynamic simulations were then conducted on these models under the canonical ensemble (NVT) in 1 fs time steps over a timespan of 200 ps, until the system reached equilibrium. Additional 50 ps MD simulations were then conducted for the actual analysis. Snapshots recorded before and after the MD simulations clearly show significant transformation of these molecular structures and their adsorption on the Au (111) surface after the MD simulations. The adhesion between Au and the buffer molecules can be evaluated on the basis of the interaction energy between the Au crystallographic surfaces and the buffer molecules. Table 2 lists the interaction energies between buffer molecules and the Au surface, as calculated using Eq. (1):

**Table 2:** Interaction energies between Au NPs and surfactants (units: kcal · mol^−1^)

Models	*E*_total_	*E*_Au_	*E*_sufactant_	*E*_interaction_
Au-PIPES	−2007.217	−1738.508	−184.083	−84.626
Au-MES	−1898.38	−1739.591	−103.473	−55.316
Au-PIPES SS	−1786.62	−1739.174	19.564	−67.01
Au-TAPSO	−1824.456	−1739.76	−24.486	−60.21
Au-TAPS	−1937.396	−1738.749	−119.521	−79.126
Au-TES	−1854.747	−1738.22	−58.735	−57.792
Au-TEA	−1777.972	−1740.113	5.0987	−42.958
Au-Bicine	−1728.022	−1740.200	−61.392	−49.214
Au-BTM	−1776.327	−1739.937	9.235	−45.625
Au-BTP	−1719.565	−1738.734	86.598	−67.429
Au-PSTT	−1750.056	−1741.751	30.412	−38.717

1 $$ {E}_{\mathrm{interaction}}={E}_{\mathrm{total}}-\left({E}_{\mathrm{Au}}+{E}_{\mathrm{reagent}}\right) $$

where *E*_Au_ is the energy of the Au surface, *E*_reagent_ is the energy of the reagent molecules, and *E*_total_ is the energy of the Au surface with the organism. Note that high interaction energy indicates high adhesive strength between the reagent and the Au surface and a more stable model in general. The interaction energies between Au and the reagents were calculated using the COMPASS force field as shown in Table 2. The simulation results produced negative values for the interaction energies of all systems, clearly indicating that the reagents were easily combined with the surface of the Au crystal. Moreover, among all models, the interaction energy of the PIPES-Au model was the highest, indicating that the strongest interaction existed between PIPES and the Au (111) surface. The results of these MD simulations provide strong evidence to help explain the relationship between the reduction of Au NPs by different reagents and the stability of these formed NPs. In particular, both theoretical and experimental observations agreed on the long-term stability of Au NPs formed using PIPES buffer.

### Biocompatibility of Au NPs

**Fig. 7:**
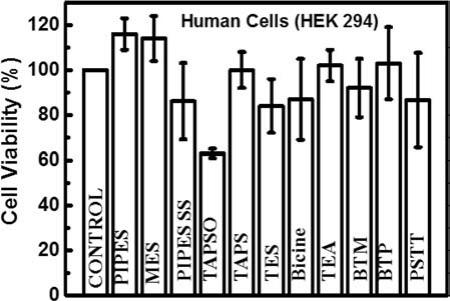
Cell viability of Au NPs in HEK 294 cells

In the MTT assay, after 24 h of post-treatment, HEK 294T cells showed excellent viability with most Au NPs, except for those produced from the reaction with TAPSO (60 %) (Fig. 7). Because of the acidic nature of TAPSO-Au, which is most stable at a pH of 2–4 (Fig. 5), it demonstrated less cell viability than the other NPs. In our observations, most of the Au NPs show small variations in their cell viability test results but were all generally non-toxic in cell tests.

## Conclusions

The present work investigated one-step syntheses of Au NPs using common biological buffers. The synthesized Au NPs were characterized using TEM, dynamic light scattering, UV-vis spectroscopy, and electrochemical experiments. Theoretical investigations were also performed using molecular dynamic simulations in order to understand the interaction between the reagents and the Au surface. The results revealed that Au NPs synthesized using the PIPES buffer have the highest zeta potential and highest interaction energy, producing the most stable NPs of the group. An MTT assay was performed to check cytotoxicity of Au NPs on HEK 293 cells, which indicated lack of any noticeable toxicity of NPs. Such Au NPs can provide new opportunities for safe and convenient applications in molecular imaging, drug delivery, therapy, and biosensors.

## Additional file

Additional file 1:
**Figures S1–S4.** Far View TEM image of Au NPs synthesized by MES buffer: **Figure S1.** Far View TEM image of Au NPs synthesized by bis-tris Methane buffer: **Figure S2.** Far View TEM image of Au NPs synthesized by Triethanolamine buffer: **Figure S3.** TEM images of unreacted buffer salts: **Figure S4**. (DOCX 575 kb)
