# Supplementary material for: Synthesis of Gold Nanoparticles with Buffer-Dependent Variations of Size and Morphology in Biological Buffers
Source: Nanoscale Res Lett. 2016 Feb 4;11:65. doi: 10.1186/s11671-016-1290-3 (PMC4742461; doi:10.1186/s11671-016-1290-3)
Supplement: Additional file 1: — Figures S1–S4. Far View TEM image of Au NPs synthesized by MES buffer: Figure S1. Far View TEM image of Au NPs synthesized by bis-tris Methane buffer: Figure S2. Far View TEM image of Au NPs synthesized by Triethanolamine buffer: Figure S3. TEM images of unreacted buffer salts: Figure S4. (DOCX 575 kb) [file 11671_2016_1290_MOESM1_ESM.docx]

**Supporting Information**

Synthesis of gold nanoparticles with buffer-dependent variations of size and morphology in biological buffers

Syed Rahin Ahmed^1,‡^, Sangjin Oh^2,‡^, Rina Baba^3^, Hongjian Zhou^4^, Sungu Hwang^5^, Jaebeom Lee^2,^^[[1]](#footnote-1)^*, and Enoch Y. Park^1,6,*^

^1^ Research Institute of Green Science and Technology, Shizuoka University, 836 Ohya Suruga-ku, Shizuoka, 422-8529, Japan

^2^ Department of Cogno-Mechatronics Engineering, Pusan National University, Busan, 46279, Korea

^3^ Department of Applied Biological Chemistry, Shizuoka University, 836 Ohya Suruga-ku, Shizuoka 422-8529, Japan

^4^ Institute of Solid State Physics, Chinese Academy of Sciences, Hefei, 230031, P. R. China

^5^ Department of Nanomechatronics Engineering, Pusan National University, Miryang 627-706, Korea

^6^ Graduate School of Science and Technology, Shizuoka University, 836 Ohya Suruga-ku, Shizuoka 422-8529, Japan.

**Keywords:** Gold nanoparticles, Good’s buffer, Synthesis route, MD simulation, Cell viability


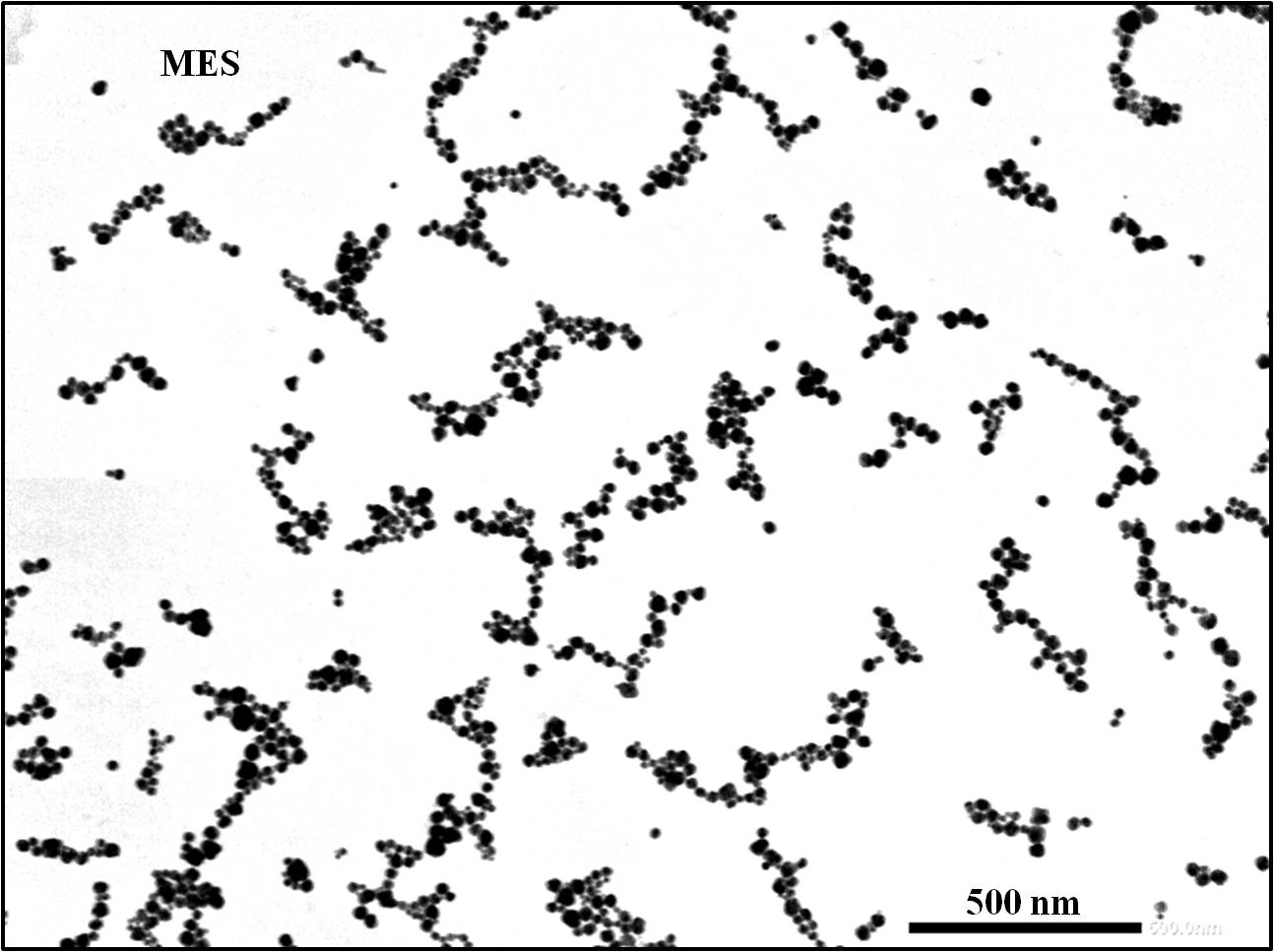


**Figure S1:** Far View TEM image of Au NPs synthesized by MES buffer.


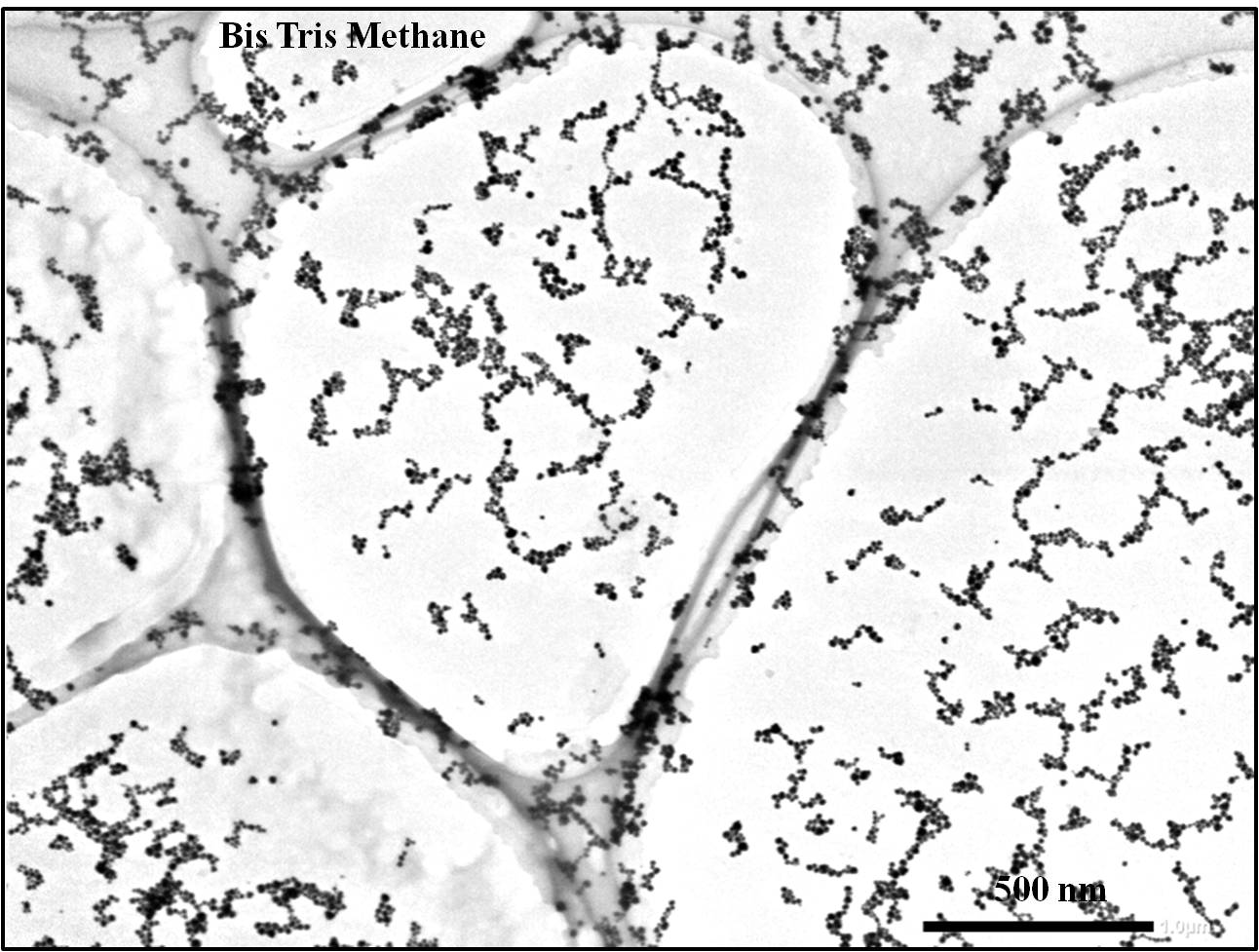


**Figure S2:** Far View TEM image of Au NPs synthesized by Bis Tris Methane buffer.


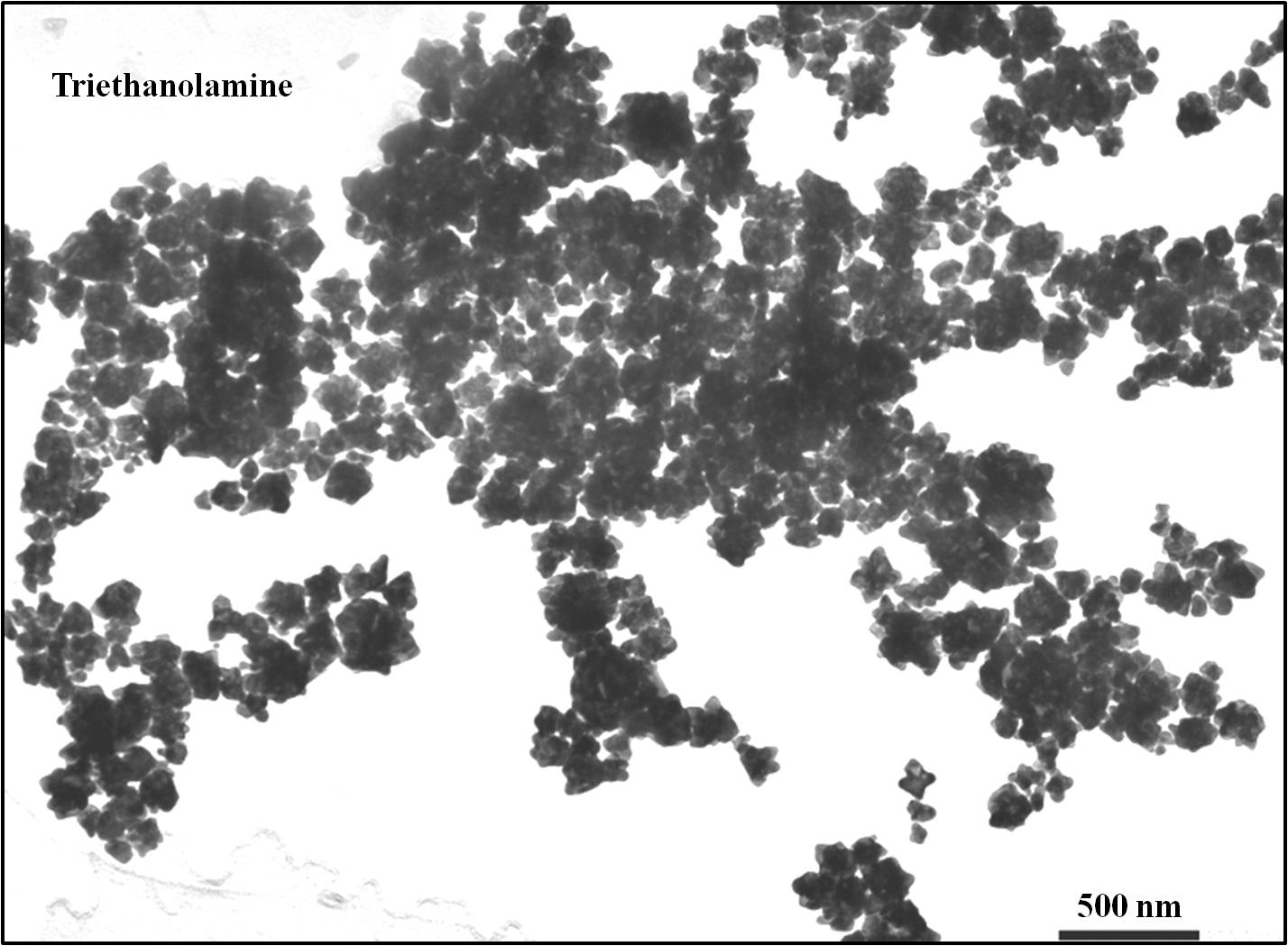


**Figure S3:** Far View TEM image of Au NPs synthesized by Triethanolamine buffer.


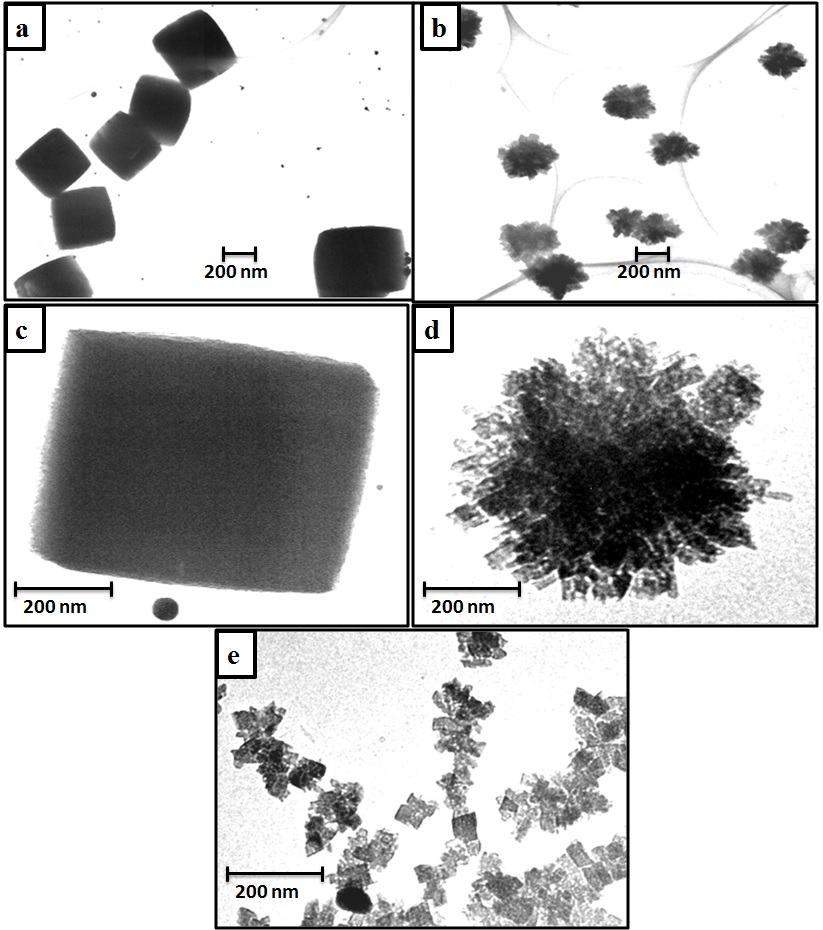


**Figure S4:** TEM images of unreacted buffer salts.

1. *Corresponding authors: jaebeom@pusan.ac.kr (JL), park.enoch@shizuoka.ac.jp (EYP)

   ^‡^Both contributed equally. [↑](#footnote-ref-1)
